# From healing and martial roots to global health practice: reimagining Tai Chi (Taijiquan) in the modern public fitness movement

**DOI:** 10.3389/fpubh.2025.1677470

**Published:** 2025-09-02

**Authors:** Dinghua Liu, Bo Zhou, Zhixiang Wen, Yang Zhang

**Affiliations:** ^1^College of Physical Education, Hunan Normal University, Changsha, China; ^2^Independent Person, Windermere, FL, United States

**Keywords:** aging, digital health, exercise, fall prevention, fitness, physical activity

## 1 Introduction

Tai Chi, referred to as Taijiquan in Chinese, embodies a unique cultural legacy that bridges ancient Chinese medical thought, martial strategy, and philosophical harmony. Born out of the dual currents of Traditional Chinese Medicine and martial necessity, it has evolved into a globally practiced method of exercise and mindfulness. In this opinion article, we propose that Tai Chi is not merely a relic of the past but a culturally rooted, adaptable, and inclusive fitness modality with increasing relevance for public health promotion across the lifespan. This reflection aligns with current calls to reintegrate culturally embedded physical practices into modern health policy and community-based intervention design (1, 2).

In light of the global burden of physical inactivity and rising chronic diseases, especially among aging populations (3), the urgency to explore movement modalities that are not only effective but also socially and culturally sustainable has never been greater. Tai Chi offers more than physical benefits; it embodies a life philosophy that promotes emotional regulation, social cohesion, and harmony with nature—elements increasingly recognized in holistic health frameworks. As more countries struggle with health disparities, there is a growing interest in accessible, low-cost interventions that provide both physical and psychosocial benefits, positioning Tai Chi as a compelling candidate for public health integration.

## 2 Healing and martial origins

Historically, Tai Chi emerged during the late Ming and early Qing dynasties, developing from earlier Daoist breathing and movement practices, and evolving into a coherent martial art by Chen Wangting in the 17th century. Its structure reflects the integration of medical qigong, battlefield defense techniques, and philosophical doctrines into a single internal martial art. Over time, different styles—including Chen, Yang, Wu, Sun—emerged. Nonetheless, Tai Chi is founded on a yin–yang cosmology, not only in its flowing movements but in its therapeutic principles. Traditional Chinese Medicine, particularly the concepts of qi, meridians, and internal regulation, informed the early structure of Tai Chi as a health-preserving practice (4). Simultaneously, its martial components were derived from ancient combat systems, informed by tactical texts such as Sun Tzu's *The Art of War* (5). Tai Chi embodies the balance of softness and strength, yielding and force (6), not merely as physical tactics, but as a philosophy for healthful living.

The yin–yang principle in Tai Chi is not a metaphor; it governs the shifting interplay of motion and stillness, expansion and contraction, attack and retreat. When practicing, the practitioner alternates between weight shifts, rotational spirals, and centering forces that mirror internal organ regulation and emotional equilibrium. This integration of intention, breath, and form forms the physical expression of Chinese cosmology. Moreover, Tai Chi's martial applications, including push hands (tuishou), joint locks, and neutralization techniques, underscore its original function as a defensive art rooted in battlefield pragmatism. These combat strategies, when examined through a modern lens, mirror core principles of biomechanics, proprioception, and reactive balance (7). This integration of martial precision and therapeutic regulation is rare among traditional exercise systems and may explain why Tai Chi has retained clinical utility in contemporary rehabilitation sciences.

This dual origin enabled Tai Chi to serve both defensive and rehabilitative functions. Breathing, posture, and internal awareness coalesce in practice, promoting circulation, postural control, and internal harmony (8). The historical image of the Tai Chi practitioner was not solely that of a fighter, but also of a healer and philosopher—someone who cultivated physical strength, ethical virtue, and mind–body awareness. This ethical dimension was deeply rooted in Confucian values of benevolence and restraint, Taoist ideals of naturalism and non-action, and the moral imperative of martial virtue (9). This inseparability of martial precision and therapeutic intent allowed Tai Chi to emerge as one of the few movement systems with robust historical continuity, integrating biomechanical control with energetic regulation in a coherent framework now highly relevant for rehabilitation and preventive care.

## 3 From cultural practice to public health asset

**Figure 1:**
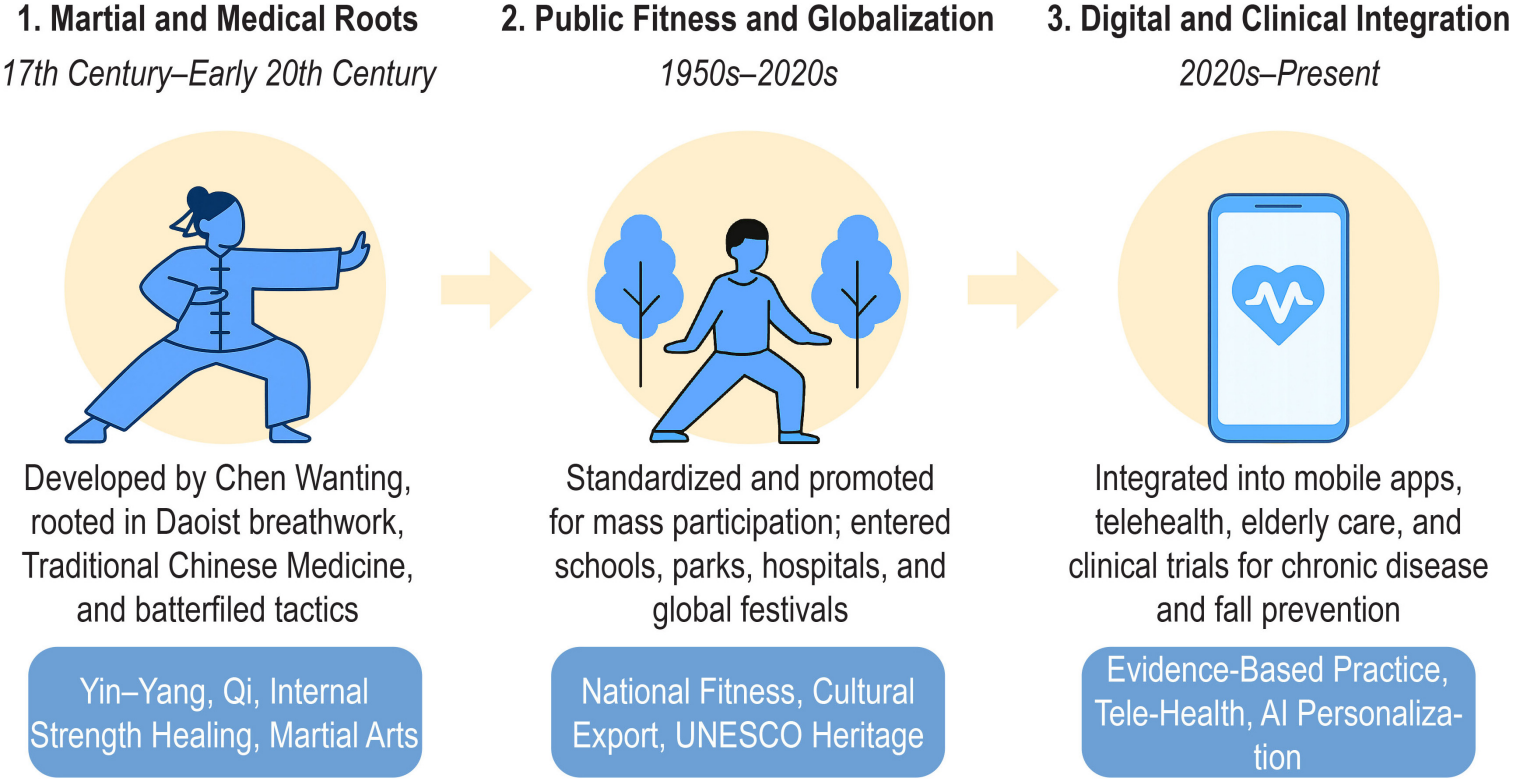
The evolution of Tai Chi: from martial origins to digital public health tool.

In the 20th century, Tai Chi underwent a profound transformation. As China transitioned through war, reform, and modernization, Tai Chi was adapted for public accessibility and stripped of its combat exclusivity. It entered parks, clinics, schools, and international festivals. This shift mirrors global trends where traditional practices are repurposed for wellness and disease prevention, as illustrated in Figure 1.

Tai Chi has been included in national fitness programs in China, and recently recognized by UNESCO as intangible cultural heritage. These efforts have supported the formal transmission of simplified forms (e.g., 24-form, 42-form Tai Chi) that can be taught to older adults, children, and people with disabilities. This democratization of access has contributed to the longevity and spread of Tai Chi globally.

Scientific evidence supports this transition from tradition to therapy. Physiological investigations have revealed a wide spectrum of health effects. Evidence from cardiovascular and pulmonary research shows that Tai Chi improves baroreflex sensitivity (10) and heart rate variability (11), markers of cardiovascular resilience. Studies have documented enhanced vagal tone and improved autonomic balance in older adults (12). Among patients with hypertension or coronary artery disease, regular Tai Chi practice has been associated with reduced blood pressure, improved lipid metabolism, and endothelial function (13, 14). In respiratory contexts, benefits have been reported among patients with chronic obstructive pulmonary disease (15), where Tai Chi enhances diaphragm strength, tidal volume, and forced expiratory measures. Musculoskeletal adaptations include improved gait speed, balance, and muscular strength (16). In a recent trial conducted by our group among fall-prone postmenopausal women, Tai Chi significantly enhanced balance control and lower limb function (17). This aligns with meta-analytic findings on improved joint mobility and reductions in fall incidence (18). Studies further suggest that long-term practitioners show gains in bone mineral density, particularly in the lumbar spine and femoral neck (19). Metabolic and inflammatory outcomes are equally promising. Tai Chi has been shown to enhance insulin sensitivity, reduce glycated hemoglobin (20), and lower systemic inflammation markers such as C-reactive protein and Tumor Necrosis Factor alpha (21). These outcomes are relevant for chronic disease prevention, particularly in the context of aging.

Evidence also supports psychological benefits. Randomized controlled trials have demonstrated moderate-to-large improvements in depressive symptoms, anxiety, and general wellbeing (22). Mechanistically, these outcomes are linked to parasympathetic activation and reductions in cortisol levels via controlled breathing and meditative movement. Clinical improvements in sleep quality have also been observed, especially in older adults and oncology patients, where Tai Chi interventions improved both subjective and objective sleep metrics (23). Further, preliminary neuroimaging studies suggest enhanced connectivity in brain regions involved in executive function and attentional control following regular practice (24, 25), offering potential value in preventing cognitive decline and mental resilience.

Beyond physical and psychological effects, Tai Chi also nurtures a sense of spiritual connectedness. Rooted in Daoist cosmology and Confucian ethics (26, 27), it fosters a meditative presence and inner harmony. Many practitioners report feelings of spiritual peace, existential grounding, and a sense of unity with nature (28–30), elements that may contribute to enhanced wellbeing and life satisfaction, especially in aging populations or those coping with chronic illness.

Taken together, this structured evidence ranging from physiological regulation to psychological resilience positions Tai Chi as an evidence-based and culturally adaptable approach for enhancing public health.

## 4 Modern relevance and global reach

Today, more than 300 million individuals practice Tai Chi globally (31), including an estimated 1.5 million in the United States alone, most of whom are non-Chinese (32). This broad uptake across continents is not merely a cultural curiosity—it reflects Tai Chi's unique ability to address universal public health concerns while transcending cultural boundaries. Its meditative movement, emphasis on balance, and low-impact nature resonate with diverse populations facing similar health challenges: aging, stress-related illness, sedentary lifestyles, and the erosion of social cohesion.

Tai Chi is particularly well-suited to fill gaps in physical activity engagement among older adults and individuals with chronic disease or mobility limitations. Unlike high-intensity fitness trends that may alienate these populations, Tai Chi offers a gentle, scalable, and sustainable alternative that aligns with global calls for age-friendly health promotion strategies. Its adaptability across cultures lies in its non-dogmatic approach: while rooted in Chinese philosophy, its practice does not require spiritual affiliation or prior cultural familiarity. Practitioners in Europe, the Americas, and Southeast Asia have incorporated Tai Chi into local healthcare systems, rehabilitation programs, senior centers, and even public school curricula (33–35). In addition to its physical benefits, Tai Chi supports broader social determinants of health. Its emphasis on group practice fosters social inclusion, intergenerational interaction, and community identity (36–38), features that are critical in societies grappling with loneliness, digital disconnection, and mental health crises. In urban environments, where access to green space is often limited and public health infrastructure strained, Tai Chi's minimal spatial and equipment requirements make it an ideal intervention for parks, rooftops, and community halls. Moreover, Tai Chi provides a counter-narrative to dominant Western fitness models that prioritize intensity, competition, and external body aesthetics. It encourages a somatic awareness that values internal balance, mindful movement, and long-term sustainability, principles increasingly recognized in trauma-informed care, integrative medicine, and mental health interventions. As such, its global relevance extends beyond clinical efficacy to embody an alternative vision of health and human flourishing.

By offering a culturally rich yet pragmatically accessible form of movement, Tai Chi has the potential to serve as a bridge between traditional wisdom and modern public health, between East and West, and between individual and collective wellbeing. Its success in international contexts demonstrates that culturally grounded practices, when supported by evidence and adapted respectfully, can play a transformative role in global health promotion.

## 5 A call to action: reclaiming cultural heritage in public health

Current physical activity frameworks often emphasize frequency, intensity, and duration as key parameters for health promotion. However, these metrics alone may overlook important sociocultural and motivational factors that determine long-term engagement. Traditional practices such as Tai Chi, while not always designed for maximal exertion, offer a contextually meaningful and sustainable form of movement that resonates with diverse populations.

To maximize Tai Chi's contribution to modern public health, future strategies should go beyond conventional clinical trials or park-based programs. The next frontier lies in leveraging digital health platforms to scale its accessibility and cultural relevance. For example, multilingual video-based programs featuring certified instructors and tailored to diverse age groups could be integrated into mobile apps, wearable ecosystems, and telehealth services. These platforms could incorporate adaptive algorithms that tailor practice intensity based on real-time biometric data (e.g., heart rate, fall risk profiles), personalized progression, and community engagement features that support adherence, particularly in rural, underserved, or socially isolated populations. Such innovations would allow Tai Chi to be practiced anywhere, reducing barriers in low-resource settings or among mobility-constrained individuals.

Real-world implementation of Tai Chi within digital and community frameworks is already underway in several regions. In mainland China, the “National Fitness Platform” app developed by the General Administration of Sport includes guided Taijiquan video modules for different age groups, with millions of registered users accessing them for daily exercise. In the United States, Tai Chi in the “Moving for Better Balance” program—endorsed by the Centers for Disease Control and Prevention—has been adopted in over 40 states, especially for fall prevention in older adults (39). Digital iterations include hybrid models like tele-Tai Chi classes via Zoom, which were widely implemented during the COVID-19 pandemic to ensure continuity in movement therapy for vulnerable populations (40). In the United Kingdom, the National Health Service has piloted community-based Tai Chi programs in public parks and GP (exercise) referral schemes as part of holistic fall prevention strategies (41). These examples illustrate not only feasibility but also sustained adherence and measurable health outcomes, providing practical models for broader integration.

Building on these efforts, cross-cultural adaptation studies are also essential. Rather than treating Tai Chi as a static tradition, researchers and practitioners should explore how local contexts can reshape its instructional language, delivery format, and group dynamics while preserving core principles. Comparative studies examining Tai Chi alongside conventional aerobic or resistance training can clarify its differential effects on mental health, neuromotor coordination, and immune function.

Moreover, governments and global health agencies could support pilot programs that blend traditional movement with modern metrics, such as heart rate variability, sleep monitoring, and fall risk assessment. These data-driven initiatives can generate the translational evidence needed to legitimize and integrate Tai Chi into policy-supported health promotion models.

In short, reclaiming Tai Chi as a living, evolving cultural asset requires that we do not simply advocate for its use—we must innovate how it is delivered, evaluated, and contextualized for contemporary global needs.

## 6 Discussion

Tai Chi's journey—from ancient battlefield and healing hall to global public parks—is a testament to its adaptability and enduring relevance. As we seek innovative ways to promote lifelong physical activity, especially among aging and marginalized populations, culturally meaningful practices like Tai Chi should not be overlooked. The legacy of Tai Chi rests not just in its techniques but in its worldview: that strength lies in flexibility, that health arises from balance, and that wellness is achieved not through domination but through harmony. In a fragmented and fast-moving world, Tai Chi invites us to slow down, reconnect with our bodies, and cultivate equilibrium—within ourselves, our communities, and the natural world. By reintegrating such practices into our public health systems, we do not merely preserve cultural heritage; we actively expand the definition of fitness to one that is more human-centered, inclusive, and sustainable. This is the future Tai Chi points us toward: one in which health is not imposed, but harmonized. The synthesis of empirical validation and cultural relevance embodied in Tai Chi offers a scalable, culturally grounded strategy to support inclusive, community-based public health solutions worldwide.
